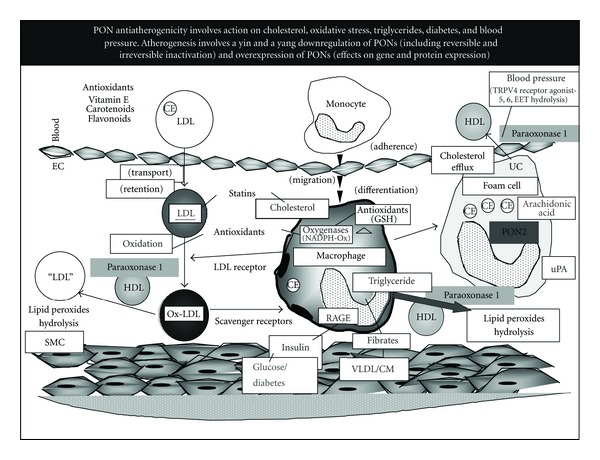# Introduction to Paraoxonases

**DOI:** 10.1155/2012/687273

**Published:** 2012-06-12

**Authors:** Michael Aviram

**Affiliations:** The Lipid Research Laboratory, Rambam Medical Center, Bat-Galim, 31096 Haifa, Israel

## 

The objectives of this special issue on paraoxonases (PONs) are to bring the latest aspects of paraoxonases (PON 1, 2, 3) research on the genetics, biochemistry, cell biology, and structural biology of PONs. The issue also addresses the role of PONs in human diseases (cardiovascular, cancer, renal failure, and gastrointestinal disorders). Inflammatory and oxidative stress-related diseases, such as diabetes or rheumatoid arthritis are also favorably affected by PON. PONs also provide microbial protection by hydrolyzing bacterial quorum lactone. 

The pivotal role of the PON family in a variety of inflammatory diseases, and in preventing the toxicity of organophosphorus insecticides and nerve agents, has made PONs an interesting target for both clinicians and scientists alike. Research into the paraoxonase family of enzymes has increased dramatically, especially following the initiation of the paraoxonase conferences. Five international conferences on paraoxonases were organized between 2004 and 2012, where the PON scientific community gathered to discuss PONs research achievements and future scientific directions. These meetings took place in Ann Arbor (MI), Debrecen (Hungary), Los Angeles (CA), La Pineda (Spain), and Columbus (OH). According to PubMed, only few PON papers were published before 1980. From 1980 till these days, about 3000 papers were published.

The PONs gene cluster contains three adjacent gene members, and all of the three PON genes share high sequence, indentify a similar *β* propeller protein structure, and hydrolyze esterase/lactonase activities.

PONs play a clear protective role against cardiovascular diseases. Major cardioprotective PON characteristics include, beside their potent antioxidant properties, the following: for PON 1, favorable effects on macrophage cholesterol metabolism, for PON 2, attenuation of macrophage triglyceride accumulation, and for PON 3, improvement in bile acids metabolism.

Human serum HDL-associated paraoxonase (PON1) is an esterase that possesses cardiovascular protective properties which result in the following antiatherogenic functions: (1) attenuated oxidative stress in serum, in lipoproteins, in macrophages, and in atherosclerotic lesions; (2) decreased oxidized LDL uptake by macrophages; (3) inhibited macrophage cholesterol biosynthesis rate; (4) stimulated HDL-mediated cholesterol efflux from macrophages.

Major PON1 inactivators (and reversal of their action) include (1) oxidative stress (and reversal effect by antioxidants such as the pomegranate polyphenolic tannin punicalagin); (2) high cholesterol (and reversal effect by statins); (3) high triglycerides (and reversal effect by fibrates); (4) high glucose (and reversal effect by insulin).

Macrophage PON2 regulation (as related to atherogenesis) differs from that of serum PON 1 in the following characteristics: (1) stimulation (not inhibition as shown for PON1) by oxidative stress and also by anti oxidants (like PON1); (2) stimulation by high glucose (PON1 is inhibited) and also by insulin (like PON1); (3) stimulation by urokinase plasminogen activator (uPA); (4) stimulation by high triglycerides (PON1 is inhibited); (5) stimulation by arachidonic acid and also by its product prostaglandin E2 (like PON1); (6) inhibition by high cholesterol and reversal effect by statins (like PON1).

Figure 1 summarizes our current view on the antiatherosclerotic properties of circulatory HDL-bound PON1 and those of cellular PON2. Reduction in specific oxidized lipids in the blood, in arterial lipoproteins (LDL, HDL), and in macrophages, by overexpressing humoral PON1 (and also cellular PON2), could thus be important target for the attenuation of atherosclerosis development.

*Michael Aviram* *Michael Aviram*

## Figures and Tables

**Figure 1:**